# Bilateral luxatio erecta: a case report

**DOI:** 10.1093/jscr/rjaf677

**Published:** 2025-08-29

**Authors:** Nicholas Frappa, Ellen Lutnick, Matthew Binkley

**Affiliations:** Jacobs School of Medicine and Biomedical Sciences, 955 Main Street, Buffalo, NY 14203, United States; Department of Orthopaedic Surgery and Sports Medicine, University at Buffalo, Jacobs School of Medicine and Biomedical Sciences, 955 Main Street, Buffalo, NY 14203, United States; Department of Orthopaedic Surgery and Sports Medicine, University at Buffalo, Jacobs School of Medicine and Biomedical Sciences, 955 Main Street, Buffalo, NY 14203, United States

**Keywords:** luxatio erecta, bilateral luxatio erecta, shoulder dislocation, inferior shoulder dislocation, bilateral shoulder dislocation, rotator cuff repair, reverse shoulder arthroplasty, cubital tunnel syndrome

## Abstract

Luxatio erecta is a rare form of glenohumeral dislocation in which the humeral head is displaced inferior to the glenoid, accounting for fewer than 0.5% of all shoulder dislocations; thus, bilateral involvement is exceedingly rare. We present a rare case of bilateral luxatio erecta in a 59-year-old male complicated by progressive ulnar neuropathy and rotator cuff deficiency, successfully managed with staged surgical intervention in the form of left-sided rotator cuff repair and right-sided reverse total shoulder arthroplasty, demonstrating good functional outcomes at over 2-year follow-up. This case adds to the limited literature related to bilateral luxatio erecta and suggests that early identification and staged management of any associated pathology may help optimize long-term functional recovery.

## Introduction

Luxatio erecta is a rare injury resulting in humeral head dislocation inferior to the glenoid, typically from forced hyperabduction [1, 2]. It accounts for fewer than 0.5% of all shoulder dislocations; bilateral involvement is exceedingly rare [2, 3]. Patients typically present with the involved arm in a fixed overhead abducted position [1, 3]. Prompt reduction is critical. Sixty percent of bilateral cases involve neurologic symptoms which typically resolve spontaneously; however, persistent neuropathy is exceptionally rare [2, 4–6]. Concomitant shoulder injuries often necessitate surgical intervention [2, 5, 7, 8].

We present a case of bilateral luxatio erecta complicated by progressive ulnar neuropathy and rotator cuff deficiency, successfully managed with staged surgical intervention, highlighting the complex recovery trajectory of this injury. The patient did consent to publication of the following case.

## Case report

**Figure 1:**
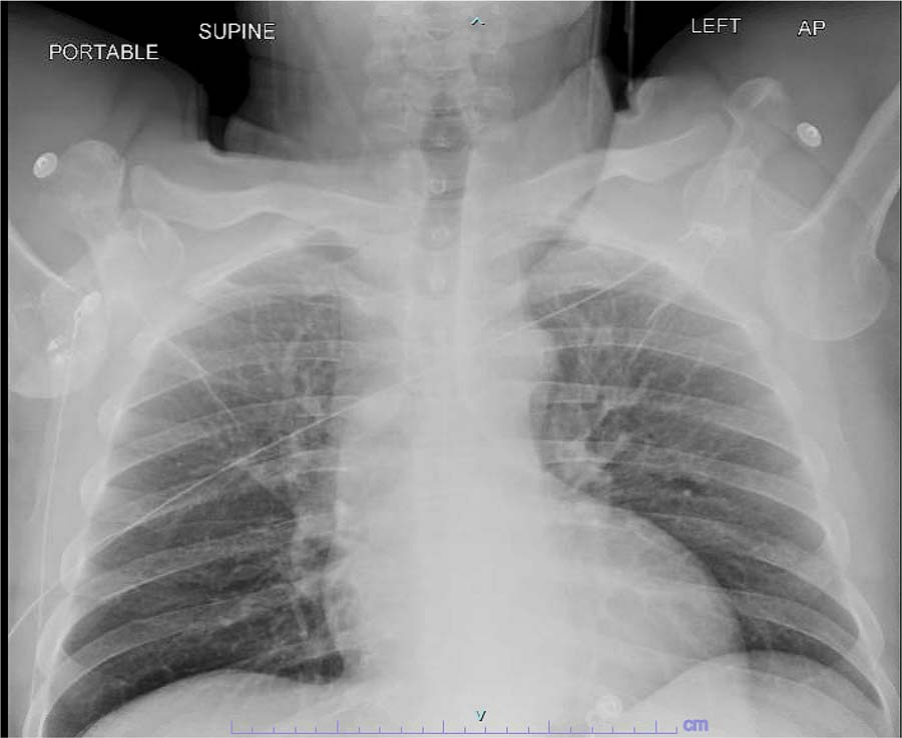
Initial chest X-ray demonstrating bilateral luxatio erecta.

A 59-year-old right-hand–dominant male with history of prior right rotator cuff repair presented to the emergency department after a workplace injury in which four 60-lb pallets fell onto his upper back, while he was operating the steering wheel of a pallet jack. He arrived with both upper extremities locked in overhead abduction, reporting bilateral shoulder pain and hand paresthesias (Fig. 1).

**Figure 2:**
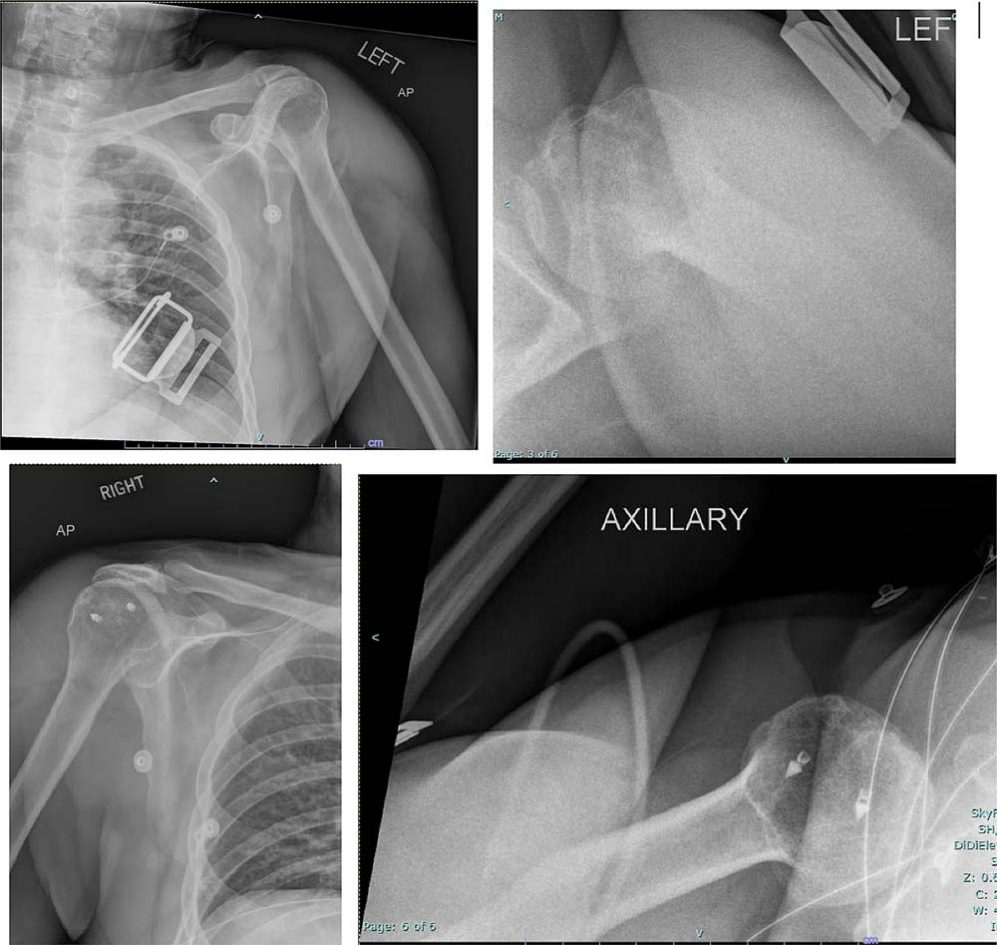
Post-reduction X-rays demonstrating congruency restored to right and left glenohumeral joints, with evidence of superior humeral head migration bilaterally.

**Figure 3:**
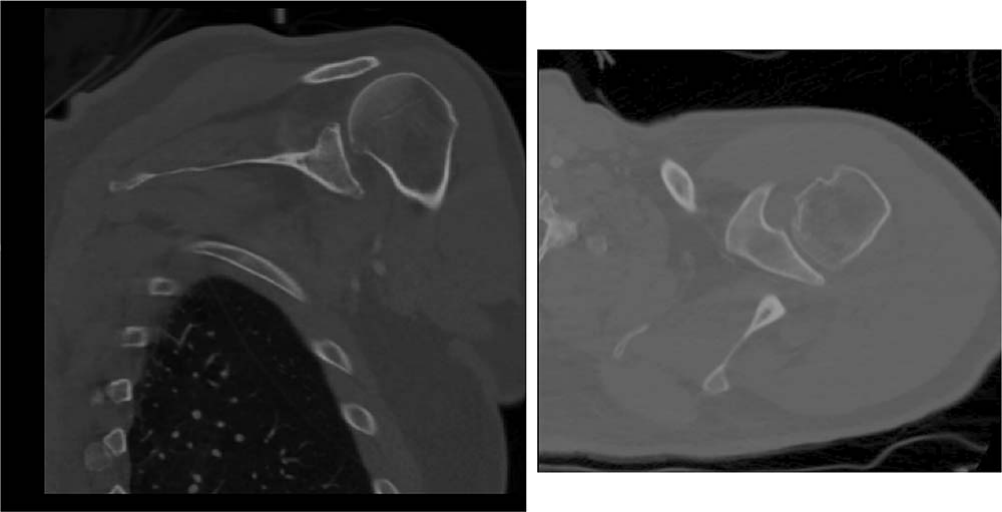
Post-reduction CT of the left shoulder demonstrating superior humeral head migration relative to the glenoid.

**Figure 4:**
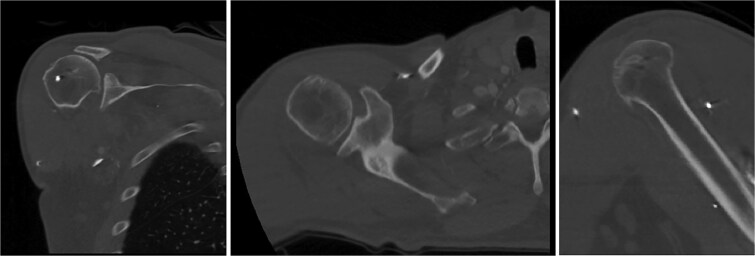
Post-reduction CT of the right shoulder demonstrating superior humeral head migration relative to the glenoid, visualized evidence of prior rotator cuff repair surgery, and a nondisplaced anterior-inferior glenoid fracture.

Both shoulders were reduced urgently by the orthopedic team, with immediate improvement in paresthesias (Fig. 2). Post-reduction CTs were significant for bilateral chronic rotator cuff disease with superior migration of the humeral heads, right-sided supraspinatus and subscapularis atrophy, and a left nondisplaced glenoid fracture (Figs 3 and 4). He was discharged home in bilateral slings.

**Figure 5:**
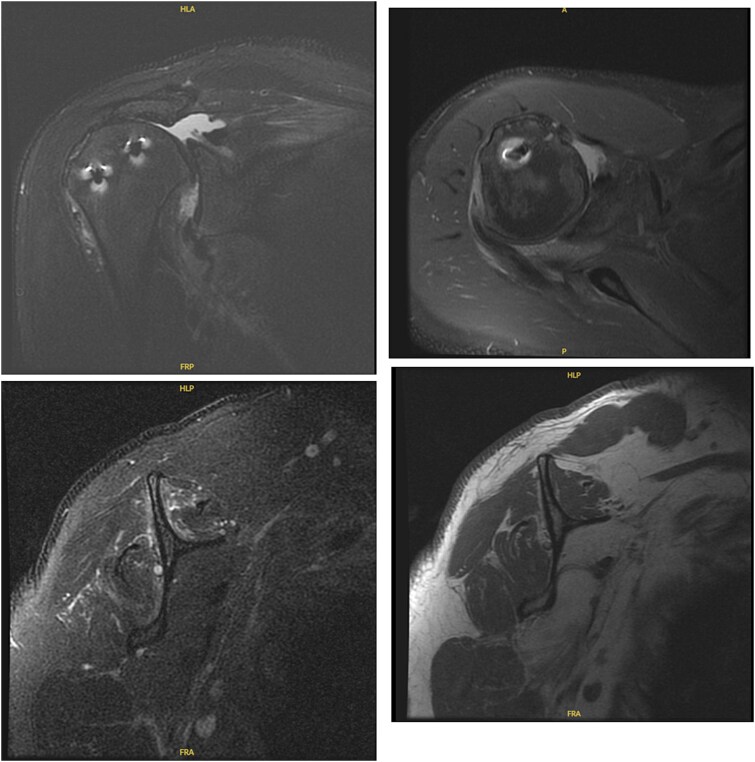
MRI of the right shoulder demonstrating full-thickness tearing of the supraspinatus and infraspinatus with medial tendon retraction, a high riding humeral head, intermediate grade partial thickness tear of the teres minor, severe fatty atrophy of the subscapularis muscle belly, labral degeneration, and tearing, partial thickness tearing of the inferior glenohumeral ligament, anterior-inferior glenoid subchondral marrow edema, and severe glenohumeral cartilage abnormalities.

**Figure 6:**
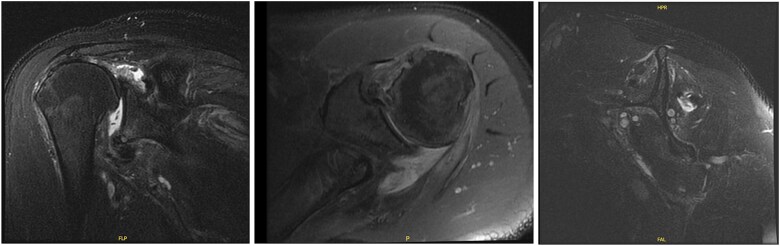
MRI of the left shoulder demonstrating a full-thickness tear of the supraspinatus and infraspinatus with tendon retraction, high grade partial thickness tearing of the subscapularis, full-thickness tear of the biceps long head tendon, complex anterior labrum tear, partial thickness tear of the inferior glenohumeral ligament including the anterior and posterior bands, inferior glenoid subchondral marrow edema, and moderate glenohumeral degenerative changes.

At 2-week follow-up, he reported continued shoulder pain. His right-sided paresthesias had resolved; however, he reported continued paresthesias into his left forearm and hand. He had significant bilateral limitation in range of motion, and weakness on internal and external rotation, more so on the left. Special tests included a positive Jobe test, drop arm test, belly press test, Obrien’s test, and Speed’s test bilaterally, as well as a positive left-sided bear hug test. He was advised to start gentle range of motion, avoid lifting, and was sent for bilateral shoulder MRIs (Figs 5 and 6).

**Figure 7:**
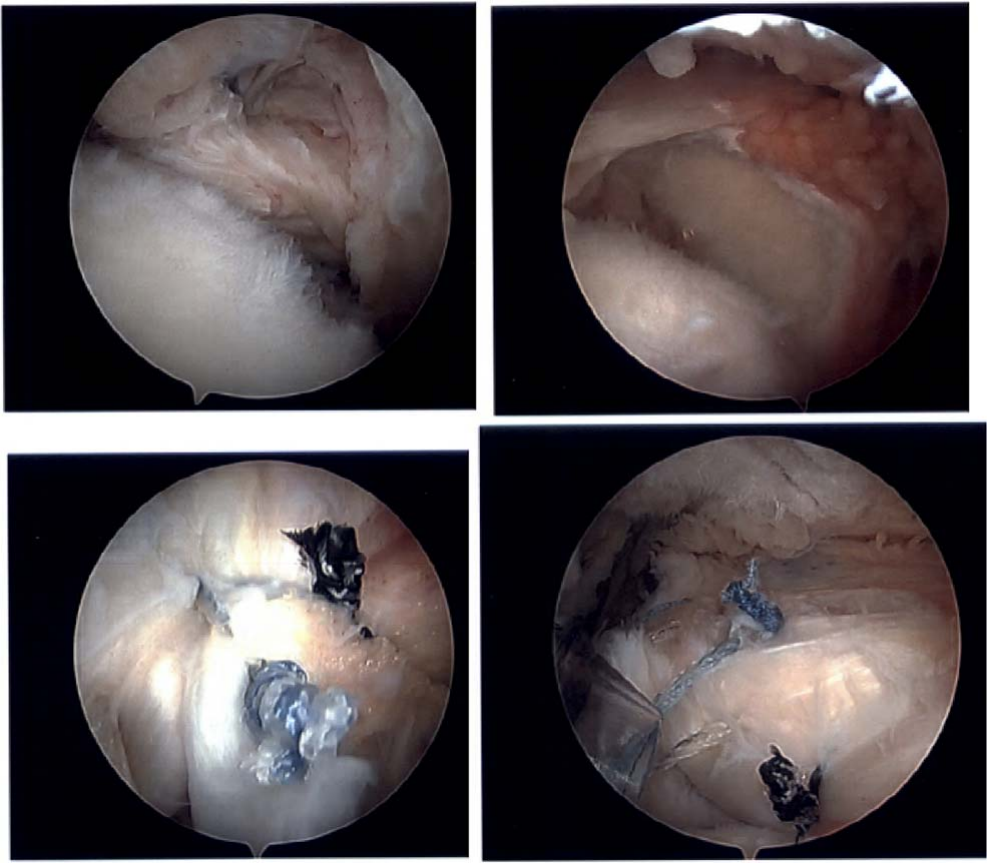
Intraoperative imaging of left shoulder arthroscopic debridement and rotator cuff repair including findings of full-thickness supraspinatus and subscapularis tendon tears, which subsequently were repaired.

The patient was determined to be a candidate for surgical intervention given his age, ongoing symptoms, and continued dysfunction. Expectations of surgical treatment were discussed, including the possibility of a less reliable outcome regarding his right shoulder given his previous surgical history. Six weeks after injury, the patient underwent left shoulder rotator cuff arthroscopic repair. Findings included full-thickness tears of the subscapularis, supraspinatus, and infraspinatus, with glenohumeral chondromalacia (Fig. 7). He was maintained in a Frank Stubbs immobilizer for 6 weeks and then started physical therapy.

**Figure 8:**
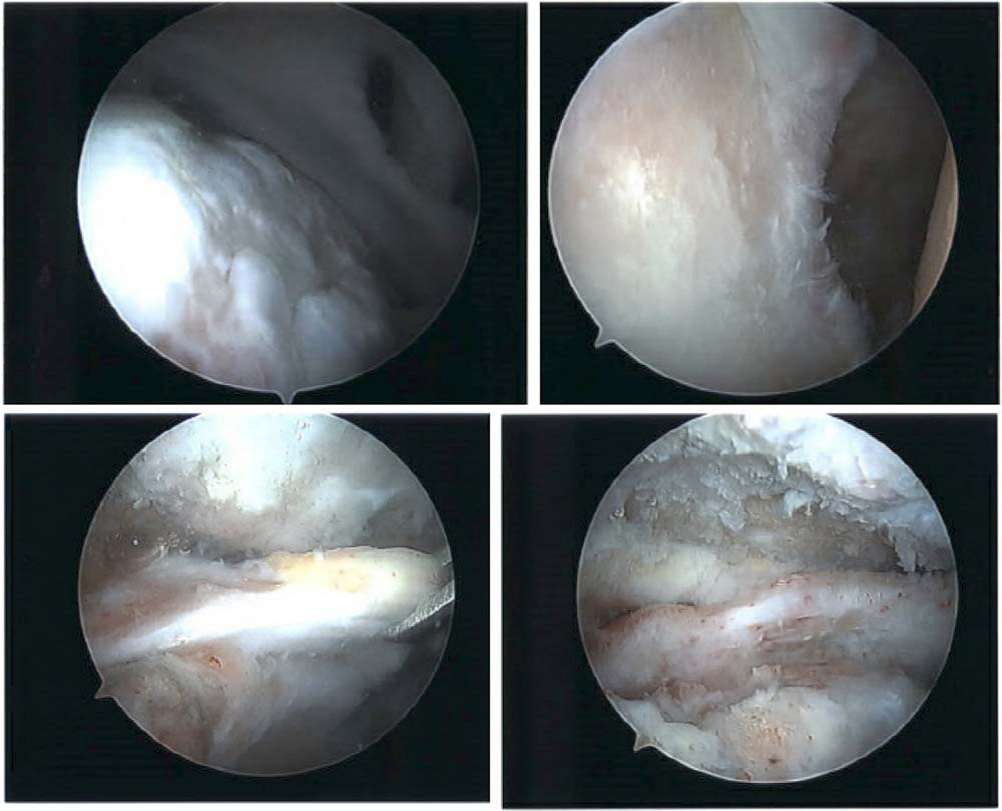
Intraoperative imaging of right shoulder arthroscopic debridement including findings of full-thickness supraspinatus and subscapularis tendon tears, which subsequently were not able to be repaired, as well as significant glenohumeral degenerative change.

The patient continued to experience right shoulder disability, including pain, limited range of motion, and sleep disturbance. Operative intervention was delayed due to elevated liver enzymes, attributed to chronic acetaminophen use; he underwent right shoulder arthroscopy ~9 months after injury. Intraoperative findings confirmed extensive, irreparable tearing of the supraspinatus and infraspinatus, glenohumeral degenerative change, and deficient biceps tendon (Fig. 8). The joint was debrided and staged reverse total shoulder arthroplasty (rTSA) was discussed.

Concurrently, the patient experienced worsening paresthesias in the left ulnar distribution, including symptomatic hand weakness. Electromyography confirmed severe cubital tunnel syndrome. Sixteen months after injury, he underwent left ulnar nerve decompression with nerve transposition. His grip strength improved, and he reported reduced numbness and better functional use of the hand.

**Figure 9:**
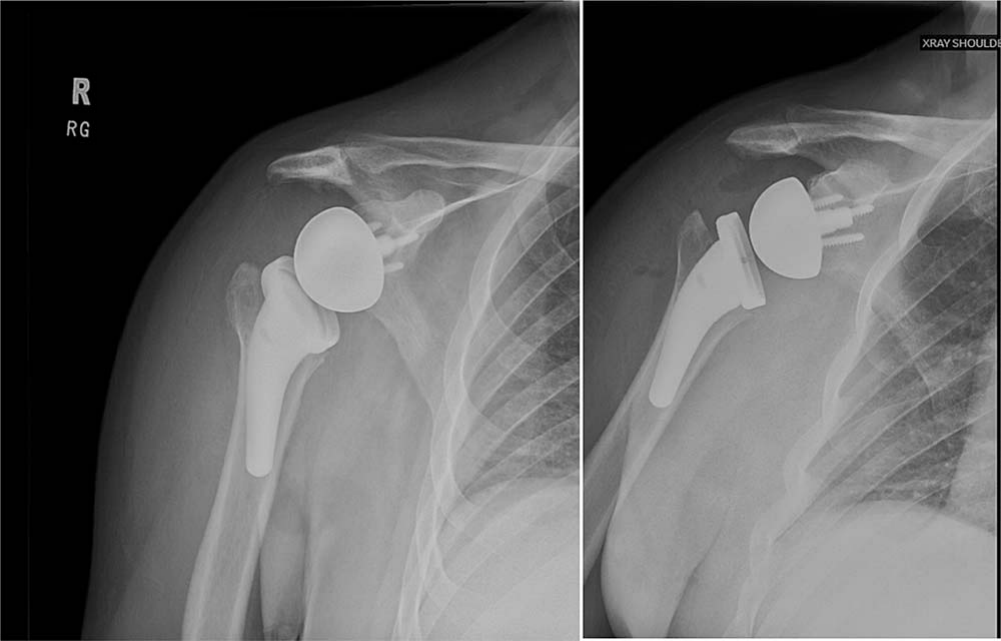
Right reverse total shoulder arthroplasty at 3-month postoperative follow-up.

At 2 years after the initial injury, he continued to have right shoulder dysfunction, with forward flexion limited to 120°, external rotation limited to 30° with his arm at his side, and notable weakness with resisted external rotation at 4/5 strength despite injections, physical therapy, and arthroscopic debridement. Ultimately, he underwent rTSA with concurrent latissimus dorsi tendon transfer to address external rotation deficits associated with his posterior cuff deficiency. The subscapularis was additionally repaired using a medialized transosseous construct. He was maintained in an immobilizer for 6 weeks and then started formal therapy (Fig. 9).

Two and a half years after injury, the patient reported continued improving function. He achieved a Disabilities of the Arm, Shoulder and Hand score of 56. Left and right shoulder range of motion measured 151° and 156° in forward flexion, 135° and 130° in abduction, and 38° and 66° in external rotation, respectively. He was independent with a home exercise program and had resumed household and outdoor activities, however, had not returned to work. His left shoulder remained more symptomatic, with activity-related discomfort and pain up to 4/10.

## Discussion

Bilateral luxatio erecta remains one of the rarest shoulder injury patterns, with just over 50 cases reported, associated with high complication rates [2]. Neurologic involvement is common, often presenting as transient paresthesias involving branches of the brachial plexus [1–3, 6]. Persistent symptoms requiring surgical intervention are uncommon. Our patient reported improved transient paresthesias after shoulder reduction; however, developed progressive paresthesias in the left ulnar distribution and declining grip strength, leading to diagnostic testing and subsequent cubital tunnel release. To our knowledge, this is the first reported case of progressive ulnar neuropathy requiring surgical decompression following bilateral luxatio erecta.

Luxatio erecta frequently results in associated injuries, including rotator cuff tears, labral injury, and glenoid or tuberosity fractures [3, 5, 9]. Our patient had bilateral chronic cuff degeneration and tearing, necessitating repair on the left and rTSA on the right. When rotator cuff repair is not feasible or fails to restore function, rTSA is an accepted treatment to restore motion and reduce pain [10]. Patients with posterior cuff deficiency may experience challenges with external rotation strength and range of motion; in such cases, a latissimus dorsi tendon transfer can be performed to improve functional external rotation [10].

Our patient ultimately achieved functional independence. However, his left shoulder remained symptomatic at final follow-up. Long-term limitations following bilateral cuff pathology or arthroplasty may be influenced by factors such as chronicity of injury, neurologic involvement, and overall functional demand [6].

## Conclusion

Bilateral luxatio erecta is an exceedingly rare injury. This case adds to the limited literature and suggests that early identification and staged management of any associated pathology may optimize long-term functional recovery.
